# Criteria for Vaginal Atrophic Changes in Genitourinary Syndrome of Menopause Using Optical Coherence Tomography

**DOI:** 10.17691/stm2025.17.2.03

**Published:** 2025-04-30

**Authors:** L.Z. Sirotina, A.L. Potapov, M.M. Loginova, L.V. Shkalova, A.D. Varnavskaya, K.V. Kazakova, A.A. Chaikin, D.A. Bolshakova, M.A. Sirotkina, T.M. Motovilova, N.D. Gladkova

**Affiliations:** Assistant, Department of Obstetrics and Gynecology; Privolzhsky Research Medical University, 10/1 Minin and Pozharsky Square, Nizhny Novgorod, 603005, Russia; Laboratory Technician, Scientific Laboratory of Optical Coherence Tomography, Research Institute of Experimental Oncology and Biomedical Technologies; Privolzhsky Research Medical University, 10/1 Minin and Pozharsky Square, Nizhny Novgorod, 603005, Russia; Junior Researcher, Scientific Laboratory of Optical Coherence Tomography, Research Institute of Experimental Oncology and Biomedical Technologies; Privolzhsky Research Medical University, 10/1 Minin and Pozharsky Square, Nizhny Novgorod, 603005, Russia; Head of Anatomic Pathology Department; Privolzhsky District Medical Center of Federal Medico-Biologic Agency of Russia, 2 Nizhnevolzhskaya naberezhnaya St., Nizhny Novgorod, 603005, Russia; 6-year Student, Faculty of General Medicine; Privolzhsky Research Medical University, 10/1 Minin and Pozharsky Square, Nizhny Novgorod, 603005, Russia; 6-year Student, Faculty of General Medicine; Privolzhsky Research Medical University, 10/1 Minin and Pozharsky Square, Nizhny Novgorod, 603005, Russia; 6-year Student, Faculty of General Medicine; Privolzhsky Research Medical University, 10/1 Minin and Pozharsky Square, Nizhny Novgorod, 603005, Russia; 6-year Student, Faculty of Pediatrics; Privolzhsky Research Medical University, 10/1 Minin and Pozharsky Square, Nizhny Novgorod, 603005, Russia; Director, Research Institute of Experimental Oncology and Biomedical Technologies; Privolzhsky Research Medical University, 10/1 Minin and Pozharsky Square, Nizhny Novgorod, 603005, Russia; Associate Professor, Department of Obstetrics and Gynecology; Privolzhsky Research Medical University, 10/1 Minin and Pozharsky Square, Nizhny Novgorod, 603005, Russia; Professor, Head of the Scientific Laboratory of Optical Coherence Tomography, Research Institute of Experimental Oncology and Biomedical Technologies; Privolzhsky Research Medical University, 10/1 Minin and Pozharsky Square, Nizhny Novgorod, 603005, Russia

**Keywords:** genitourinary syndrome of menopause, vaginal atrophy, optical coherence tomography

## Abstract

**The aim of the study** is to determine the criteria for vaginal atrophy in genitourinary syndrome of menopause (GUSM) and the age norm by comparing optical coherence tomography (OCT) data and histological findings of biopsy.

**Materials and Methods:**

The study involved 25 patients with clinical presentation of GUMS and 3 virtually healthy women (mean age — 56.7±1.4 years). On gynecological examination the patients underwent colpo- and vaginoscopy, their vaginal health index being calculated. OCT study was performed in three anatomical points of the upper vagina and the vaginal vestibule (these regions are rich in estrogen receptors and most frequently affected in GUSM). The biopsy was taken from the right point of the vaginal vestibule followed by a histological examination and PAS reaction to reveal glycogen. The epidermal thickness was quantitatively assessed by OCT and histological images. There were determined the signal levels from the epithelium and the connective tissue, the epithelial stromal stratification being performed.

**Results:**

Normal vaginal mucosa in OCT images had stratified structure including the epithelium 503 [467; 550] μm thick, with low intensity of OCT signal and the proper mucous plate with a high OCT signal. As a result of the difference in signal levels from the epithelium and the proper mucous plate, the contrast boundary formed between them. The signal level from the epithelium was 54.1 [51.5; 56.3] RU, and that from the connective tissue — 70.7 [65.9; 73.7] RU. The mucosa had folds, which in OCT images looked like a waveform boundary of the epithelium and the submucosa.

Based on vaginal health index values and colpo- and vaginoscopy findings, GUSM patients were divided into 2 groups: patients with mild mucosal atrophy and those with severe mucosal atrophy. The first group of patients was observed to have the reduced epithelial thickness of up to 261 [244; 289] μm; the signal from the epithelium increased due to the decrease in glycogen content, and was 61.6 [55.0; 65.5] RU, and the connective tissue signal was 79.5 [77.2; 79.9] RU. Mucosal folds were not visible. Severe atrophy patients had a significant decrease in the epithelial thickness, up to 158 [143; 191] μm; the signal from the epithelium was 69.7 [67.1; 72.4] RU reducing the boundary contrast of the epithelium and the submucosal base (it can be explained by glycogen absence); the signal from the connective tissue was 90.32 [80.90; 101.60] RU. Mucosal folds were not visible. The stratification index showed no changes due to the fact that the signal intensity in vaginal atrophy increases synchronically from both: the epithelium and the proper plate (stroma). The epidermal thickness measured histologically showed a high coherence level with OCT measurements (r=0.93; p<0.0001).

**Conclusion:**

The study determined OCT criteria of the age norm for vaginal mucosa changes and atrophy in mild and severe GUSM that will enable to facilitate the personalization of the therapy approaches and optimize the management of such patients.

## Introduction

Perimenopausal and postmenopausal age is one of the important periods in woman’s life. There is reproductive hormonal (including estrogens) deficiency against the background of ovarian functional depression; it is manifested by reduced general condition, as well as the local symptoms of vulval and vaginal atrophy. The clinical presentation of vulvovaginal atrophy (vaginal dryness, itching, burning; colpoptosis) combined with sexual impairments (dyspareunia, postcoital bloody discharges) and urinary disorders (hyperactive urinary bladder, urinary incontinence, recurrent urinary infections) are generally termed as genitourinary syndrome of menopause (GUSM). GUSM manifestation is rather frequent problem in women of perimenopausal age. According to various sources, from 50 to 70% of patients face the symptoms, however, far from everybody can share her problems with a gynecologist due to a feeling of embarrassment, often justifying current developments by age changes [1, 2].

The vaginal wall consists of three layers: internal — mucosa, middle — a smooth muscle layer, external — adventitious. The vaginal layer is lined with nonkeratinized glycogenized stratified squamous epithelium, and consists of four cell types: basal, parabasal, intermediate and superficial forming about 30–40 rows. Beneath the epithelium there is the proper mucous plate, which presents the connective tissue and has collagen, elastic fibers, fibroblasts, blood and lymph vessels, as well as nerve fibers. Estrogen receptors are found in the vaginal epithelium (basal and parabasal layers), smooth muscle cells of vessels, skin, perineal striated muscles, urothelium, as well as in vaginal vascular endothelium, in the wall of the urinary bladder and urethra. Estrogens affecting the receptors in stratified squamous epithelial layers stimulate cell proliferation and differentiation. The activation of estrogen receptors on blood vessels results in the mediated stimulation of nitrogen oxide production, vasodilatation, and increased microcirculation in tissues due to blood flow velocity increase and congestion decrease at the level of small vessels; when the receptors are activated, in vaginal stromal fibroblasts collagen synthesis stimulation occurs [3, 4]. Vaginal walls contain no iron, therefore, vaginal secrete forms due to the transudation from the vaginal wall vessels and also the grandular secretion of the cervical canal and the vaginal vestibule [5].

Associated with estrogen deficiency, vaginal circulation decreases, proliferative processes in the mucosa cease (the portion of superficial and intermediate cells decreases), collagen destroys, vaginal epithelium becomes thinner, the vagina loses its elasticity, and vulvovaginal atrophy develops [5]. GUSM manifestations are not always consistent with the severity of the objective signs of urogenital atrophy [6], although they cause severe psychosocial disorders and have a significant negative effect on woman’s life quality.

Currently, GUSM is diagnosed based on the past history (King’s Health Questionnaire — KHQ, the Day-toDay Impact of Vaginal Aging — DIVA, the Female Sexual Function Index — FSFI), the visual assessment of the vaginal mucosa (vaginal health index — VHI), colpo-, vagino-, and vulvoscopy, as well as the cytological screening to calculate an epithelial maturation index; in some cases, when indicated, vaginal wall biopsy can be taken with the following histological study. However, subjective data of questionnaires, gynecological examination, and laboratory findings do not always correlate with each other, consequently hampering the personalization of therapy approaches and the optimization of GUSM patient management [7]. Moreover, considering the presentation is similar to other pathological conditions, the differential diagnosis is necessary, e.g., with vaginal epithelial dysplasia, vaginal cancer. For this purpose, vaginal wall biopsy is needed to be taken for histological study to confirm the diagnosis. Notice that the procedure of taking biopsy material can cause vaginal wall bleeding and inflammation that can aggravate the patient’s symptoms.

The mentioned factors necessitate the advisability of searching for a new technique to assess the vaginal morphology, it being highly competitive with the sensitivity and specificity of a histological study, and less invasive to be able to perform periodic monitoring (e.g., in long-term ineffective treatment). Based on the anatomy of female reproductive system, it seems possible to approach the object under study (vagina) using modern technologies of optical imaging, e.g., optical coherence tomography (OCT). It is a noninvasive method using near-infrared optical radiation, which enables to take real-time high-resolution images of tissues. OCT is also called “an optical biopsy”, since the technique succeeds in pretty exactly capturing the tissue morphology based on the differences of the scattering properties of its different components [8].

OCT has already been successfully applied in diagnosing some gynecological conditions. There was assessed the endometrial condition on *ex vivo* samples and by *in vivo* OCT and elastography: there were specified the criteria, which facilitated to determine conditionally normal endometrium, hyperplasia and cancer [9–11]. OCT made it possible to differentiate normal vulval skin from the skin affected by vulval lichen sclerosis, lichen ruber planus, as well as to estimate different degrees of damage *in vivo*, including the use of OCT angiography and lymphangiography [12–14]. In case any neoplastic processes are suspected, OCT is used to assess the uterine cervix condition [15, 16]. There are the reports on studying the vaginal elastic properties in pelvic organ prolapse (the anterior and posterior vaginal wall) *ex vivo* [17, 18]. Based on the results of successful experience using OCT to diagnose a number of gynecological conditions, the method was decided to be applied to study the vaginal mucosa condition, in particular epithelium, in GUSM.

The major advantage of OCT is its usability in outpatient settings — no patient’s long-term preparation is needed that significantly eases and promotes diagnostics, as well as excludes the risks of possible invasive intervention.

**The aim of the study** is to assess the feasibility of using OCT as an objective (quantitative) diagnostic approach to intravital vaginal study to estimate the mucosa condition and reveal early and late signs of atrophic changes in GUSM.

## Materials and Methods

### Patients and research design

The vaginal mucosa was studied in Sadko Clinic (Russia) from February to December 2023 in virtually healthy women (n=3) and patients with clinical presentation of GUSM (n=15). The selection criteria for study subjects were the following: age from 45 to 70 years (mean age — 56.7±1.4 years); GUSM presentation. Exclusion criteria were: menopausal hormonal therapy and local therapy by estriol; the therapy being provided in the periods less than 3 months before entering the investigation; active systemic autoimmune processes; polyvalent allergy; acute inflammatory and oncological diseases; decompensated diabetes mellitus, large hysteromyoma, hyperplastic endometrium, ovarian tumors, untreated sexually transmitted diseases.

**Figure 1.**
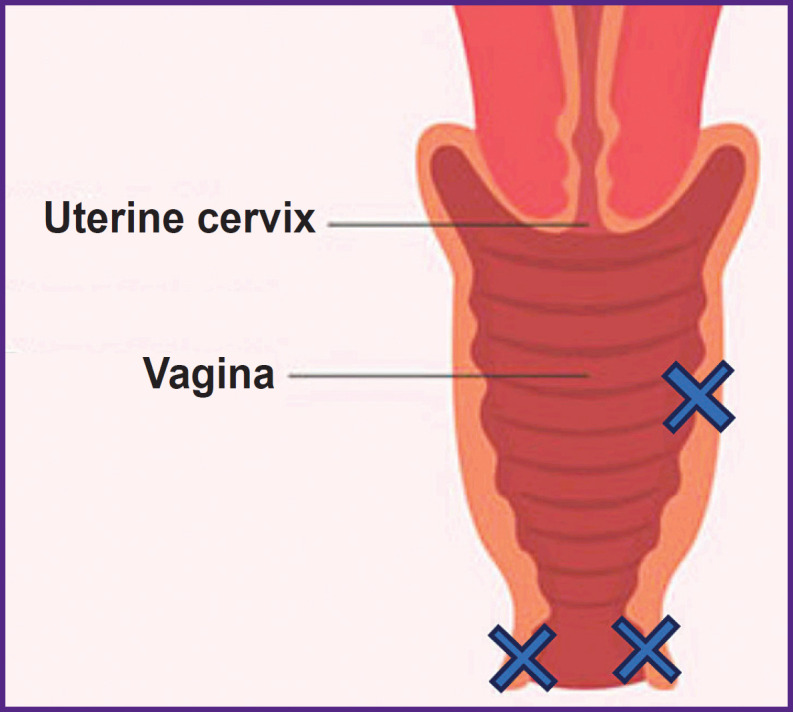
Vaginal image, coronal view Crosses mark OCT study points

The study objects were the vaginal vestibule and the upper vagina. OCT was performed in two symmetrical points (left and right) of the vaginal vestibule, and one point in the upper vaginal regions, since these parts are the most frequently affected due to high concentrations of estrogen receptors in tissues [19, 20]. The biopsy was taken once from the right point of the vaginal vestibule (Figure 1).

For clinical assessment of the vaginal condition on gynecological examination, we applied VHI using Bokhman’s scale [21]. A number of parameters were measured: the presence of discharges, vaginal pH value, the wall elasticity, the humidity degree, the epithelial condition — each of the parameters was estimated in scores. The sum of the scores was VHI value. The test gives an idea on the vaginal mucosa functioning. High scores (from 20 to 25) are generally assumed to show relatively healthy epithelial condition: 15–20 scores — mild atrophy, 15 scores and less — severe atrophic changes of the vaginal wall mucosa. Colposcopy and vaginoscopy were used to specify the epithelial condition: a visual assessment method of the epithelial layer of the vaginal wall. Colpo- and vaginoscopic atrophic signs are the following: pale thinned epithelium, the presence of subepithelial vessels, petechial hemorrhage, contact bleedings, weak staining by Lugol’s solution (positive staining intensity shows glycogen content in the epithelium). The method enables to quickly visually prove the present atrophic changes in GUSM, however, the findings of the test and stratification by severity are rather relative, it depends on doctor’s experience and skills for performing colpo- and vaginoscopy, and the subjectivity of interpreting the findings.

Despite the difficulties, the patients’ grouping concerning the atrophic severity of epithelial changes should correlate with VHI and colpo- and vaginoscopy findings. However, the histological study of a bioptate is a gold standard for assessing atrophic changes in the vaginal wall mucosa.

All patients signed an informed consent to participate in the study and the vaginal wall mucosa biopsy. The investigation was approved by the Ethics Committee of Privolzhsky Research Medical University (protocol No.4 dated March 14, 2022).

### Optical coherence tomography system

*In vivo* vaginal mucosa was studied on OCT system “ОКТ 1300-Е” (BioMedTech, Russia; certificate of registration FSR 2012/13479). The system is equipped with a flexible optical fiber probe, which ends with a ‘pencil-type’ end lens (length — 8 cm, diameter — 1 cm), and designed for contact tissue study. Secure retention of the probe in relation to the object under study is due to a special handle. OCT system operates at central wavelength of 1310 nm, bandwidth of 100 nm. The imaging rate is 20,000 A-scans/s, resolution by width — 10 μm, transverse resolution — 15 μm. The system will create a set of 3D data, 3.40×3.40×1.25 mm^3^ in size, in real-time mode, with scanning duration 26 s.

**Figure 2.**
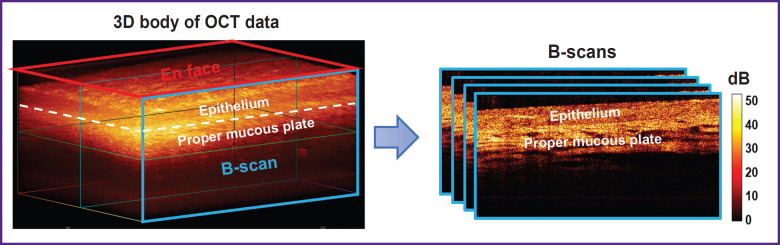
Obtaining structural images from the initial 3D body of OCT data of the vaginal mucosa

From the 3D data set, real-time structural OCT transverse images — B-scans — are obtained (Figure 2).

### OCT data analysis

The analysis consisted of three stages: 1) the visual analysis of structural OCT images to specify the features typical for mucosal atrophy; 2) the quantitative assessment of the epithelial thickness; 3) the assessment of the signal intensity from the epithelium and the proper mucous plate, the calculation of an epithelial-stromal stratification index.

The epithelial thickness was measured as the segment length from the epithelial surface to perpendicular to it the boundary of the epithelium and proper mucous plate, then a mean value for nine measurements was calculated. Then, three structural images from 3D data bulk (two outermost and central В-scan) were chosen, on each of which three measurements were taken. For measurements we used the standard functions of ImageJ program (USA).

OCT signal intensity in the epithelium and the proper mucous plate was calculated using the functions of measuring the mean grey level in the specified region of ImageJ program. When measuring OCT signal intensity in the proper mucous plate, we did not take into consideration rimiform inclusions with low signal intensity, which corresponded to lymph nodes.

An epithelial-stromal stratification index was calculated according to the formula [22]:

Stratification=I(e)−I(pr.pl.)/I(e)+I(pr.pl.),

A stratification index enables to assess the relation of the signal level from the epithelial to the signal from the proper mucous plate, and takes negative values in case of the higher signal from the proper mucous plate. The more the index deviation from zero (to positive or negative), the greater the difference of signal intensity and the contrast of the boundary of the epithelium and the proper mucous plate.

### Histological analysis

Immediately after OCT study, the incision biopsy under local application anesthesia (2% lidocaine) was taken from the right point of the vaginal vestibule to interpret the obtained OCT images. A resected sample was placed into a histologic cassette with foam lining, and embedded into 10% buffered formalin solution followed by standard histological processing, and the histological specimens were made. The preparations were stained by hematoxylin and eosin to assess the general picture of the epithelium, the proper mucous plate, connective tissue structures to characterize a vascular component and the inflammatory process components. Picro-Mallory trichrome staining was performed to assess the connective tissue condition (collagen fibers were blue stained, elastic fibers were stained — from pale pink to yellow). A histochemical PAS reaction was used to reveal glycogen in the epithelium and exclude mycotic lesions (glycogen was pink-red stained). The findings were digitized using the imaging system EVOS M7000 (Thermo Fisher Scientific Inc., USA) and described in detail by an experienced pathologist. The epidermal thickness was measured by means of morphometric functions of ImageJ program.

### Statistical analysis

The data were statistically processed using GraphPad Prism v. 9.0 (USA). The quantitative results were represented as median value with 25^th^ and 75^th^ distribution percentiles (Me [Q1; Q3]). Shapiro–Wilk test was applied to check for normal distribution, which demonstrated the data being non-normally distributed. Therefore, for statistical evaluation we used a non-parametric Mann–Whitley criterion (for paired comparisons) and Kruskal–Wallis test (for multiple comparisons) with Bonferroni adjustment. To calculate the correlation value of the vaginal mucous epithelial thickness calculated according to OCT data and histological findings, we used Spearman correlation coefficient (r). The differences were considered significant if p<0.05.

## Results and Discussion

### Relative age norm group

According to clinical assessment, the patients’ VHI was 24 [24; 25] scores, colpo- and vaginoscopy revealed no atrophy signs.

**Figure 3.**
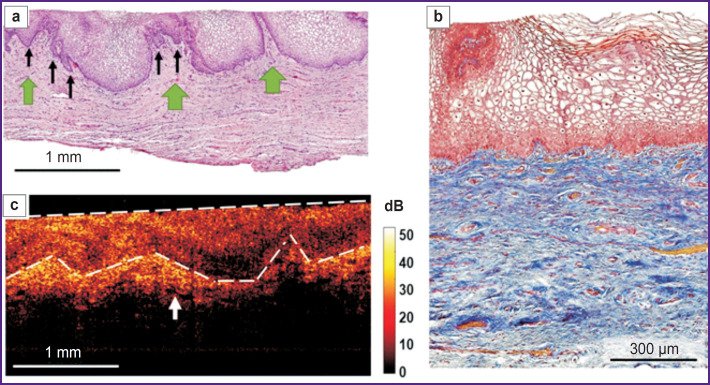
Histological and OCT study of normal vaginal vestibule mucosa: (a) a survey histological image, green arrows indicate the mucous fold, black arrows — epithelial crista, hematoxylin and eosin staining, ×40; (b) histological study of the connective tissue, Picro-Mallory staining, ×200; (c) structural OCT image (В-scan), a white dotted line delimits the epithelium, a white arrow indicates the lymphatic vessel

The histological examination demonstrated the multilayer glycogenized non-keratinized squamous epithelium consisting of 40–50 cell layers, and the underlying proper mucous plate presented by the connective tissue (Figure 3 (a)). The epithelium had crista protruding into the submucosa, the latter being crunched up having large folds. The proper mucous plate when stained by Picro-Mallory consisted of thin, incoherently arranged collagen fibers primarily oriented parallel to the epithelium (Figure 3 (b)). There were observed moderate vascular congestion and weak lymphocyte infiltration in the proper mucous plate immediately beneath the epithelium. PAS reaction revealed glycogen grains in the cytoplasm of the superficial and intermediate epithelial layers in the form of a continuous layer.

Normal mucosa in structural OCT images (Figure 3 (c)) had the layered structure: the first (upper) layer corresponded to the epithelium, and was characterized by the lower level of OCT signal; the second (lower) layer was the proper mucous plate and had the higher signal level, since it contained collagen fiber bundles, which exhibited high scattering. Structural OCT images well demonstrated the boundary of the epithelium and the proper plate, although they failed to visualize the epithelial crista. The boundary of the epithelial layer and the submucous layer had wavy structure (Figure 3 (c), *white dotted line*) due to vaginal mucous folds formed by the proper mucous plate folds. The first layer surface (epithelium) in B scans was even due to the use of a contact probe. In the proper mucous plate, there were single elongated inclusions with a low OCT signal, which were the lymph vessels (Figure 3 (c), *white arrow*).

The epithelial thickness in structural OCT images was 503 [467; 550] μm; in histological images it was 529 [462; 625] μm. The obtained epithelial thickness values in structural OCT images were comparable to those in histological images.

### Vaginal wall mucous structure in atrophy

When taking the past history, we revealed the group of GUSM patients to have a significant range of VHI values (from 5 to 19 scores), the clinical presentation of atrophy severity in colpo- and vaginoscopy and the epithelial thickness (from 100 to 400 μm) according to the histological study. Moreover, the abovementioned parameters were found to have a high correlation degree. For this reason, we divided the patient cohort into two groups: mild atrophy (n=7) and severe atrophy (n=8) patients.

In the patients with ***mild atrophic changes*** VHI was 17 [16; 20] scores, they were determined to have slight changes according to colpo- and vaginoscopy (thin pale epithelium, the moderate amount of visualized subepithelial vessels, non-uniform weak staining by Lugol solution).

**Figure 4.**
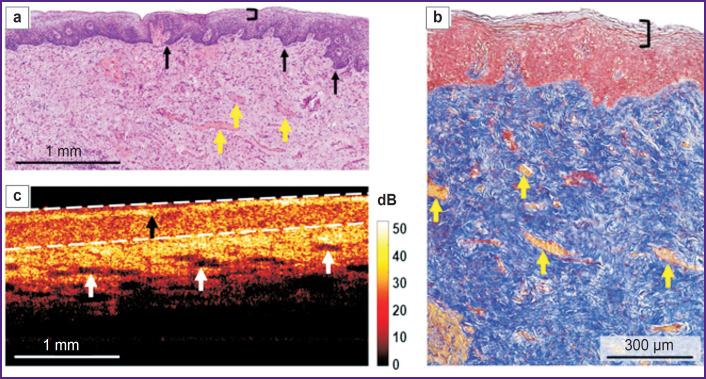
Histological and OCT study of the vaginal vestibule mucosa in mild atrophy: (a) a survey histological image, no mucous folds, black arrows indicate the epithelial crista, a black bracket restricts the horny layer with preserved nuclei (parakeratosis), yellow arrows indicate full-blood vessels, hematoxylin and eosin staining, ×40; (b) histological study of the connective tissue, a black bracket restricts parakeratosis, yellow arrows indicate full-blood vessels, Picro-Mallory staining, ×200; (c) structural OCT image (В-scan), a white dotted line delimits the epithelium, a black arrow indicates the horny layer, white arrows — lymphatic vessels

According to histological findings (hematoxylin and eosin staining), there was found the epithelial atrophy (thinning) due to the reduced number of cell layers up to 20–25, and glycogen loss; on the surface there was a layer of horny cells with preserved nuclei (parakeratosis) (Figure 4 (a)). The number of epithelial papillae was decreased, there were no proper mucous plate folds. When Picro-Mallory stained, the proper mucous plate consisted of thin, although densely arranged collagen bundles, without preferred orientation (Figure 4 (b)). There were observed marked vascular congestion and weak lymphocyte infiltration in the proper mucous plate. PAS reaction revealed glycogen grains in the cytoplasm of the intermediate layer in the form of discontinuous areas.

Structural OCT images also enabled to detect the stratified structure of the vaginal mucosa and the boundary of the epithelium and the proper plate (Figure 4 (c)). The epithelial layer was even due to no vaginal folds; and had a horny layer with a high signal (Figure 4 (c), *black arrow*). The signal from the epithelium was higher compared to the norm, it was related to both — early epithelial maturation and keratohyalin accumulation, and also the reduced glycogen amount. The epithelial thickness was reduced along the entire length of the layer. The signal penetrated into the proper plate more deeply, since it did not attenuate in the thin epithelium. The signal level from the connective tissue was higher than that in the norm, it was related to the denser and disordered arrangement of collagen bundles. There were observed numerous elongated inclusions with a low OCT signal level — the lymph vessels, which in histological examination looked collapsed and poorly defined. Marked vascular congestion enabled to conclude about intravital lymphatic bed activation.

In histological images the epithelial thickness was 261 [244; 289] μm, in structural OCT images — 239 [237; 297] μm. It also was the evidence of the comparability of the obtained data on the epithelial thickness in histological and structural OCT images.

In ***severe atrophy*** VHI was 7 [6; 11] scores, there were determined marked changes according to colpo- and vaginoscopy (pale mucosa, the presence of numerous subepithelial vessels with petechial hemorrhage, contact bleeding, weak staining by Lugol solution).

**Figure 5.**
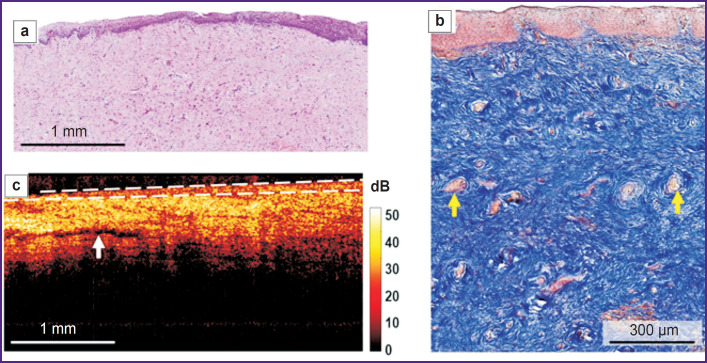
Histological and OCT study of the vaginal vestibule mucosa in severe atrophy: (a) a survey histological image, no mucous folds, severe epithelial atrophy, hematoxylin and eosin staining, ×40; (b) histological study of the connective tissue, densely arranged thin and thickened collagen bundles, yellow arrows indicate full-blood vessels, Picro-Mallory staining, ×200; (c) structural OCT image (В-scan), a white dotted line delimits the epithelium, a white arrow indicates the lymphatic vessel

According to histological examination, hematoxylin and eosin staining revealed marked epithelial atrophy (thinning) due to the reduction of cell layers up to 12– 15, nearly complete glycogen loss (Figure 5 (a)). There was a thin horny layer. There were single epithelial crista and no submucous folds. The proper plate when Picro-Mallory stained was found to consist of thin and thickened, closely arranged collagen bundles, without preferred orientation (Figure 5 (b)). There were observed the congestion of some vessels and weak lymphocyte infiltration in the proper plate. PAS reaction revealed the slight amount of glycogen grains in the form of foci.

Structural OCT images also enabled to visualize the vaginal wall layers; however, the boundary of the epithelium and the proper plate contrasted worse than in mild atrophy (Figure 5 (c)) that was related to an increased signal from atrophic, thin epithelium poor in glycogen and accumulated keratohyalin. The signal penetrated into the proper plate more deeply than in the norm, since it did not attenuate in the thin epithelium. The signal level from the connective tissue was higher than that in the norm, it was related to denser and disordered arrangement of collagen bundles. In the proper plate there were observed single elongated inclusions with a low OCT signal (lymph nodes).

In structural OCT images the epithelial thickness was 158 [143; 191] μm. The histological images showed sharp decrease in the epithelial thickness — up to 140 [101; 168] μm. The histological and OCT data were also well comparative.

All quantitative and qualitative OCT signs are represented in the summary Table. The analysis of the obtained data enables to suggest using 4 criteria (3 quantitative and 1 visual) for differential diagnosis of the vaginal condition: the epithelial thickness, OCT signal level from the epithelium, OCT signal level from the proper mucous plate, and the presence of mucous folds. The values of the stratification index and the boundary contrast of the epithelium and the proper mucous plate demonstrated no statistical significance.

**Table:** Visual and quantitative OCT parameters of normal vaginal mucosa and in genitourinary syndrome of menopause with mild and severe atrophy, Me [Q1; Q3]

Criteria	Norm	Atrophy in genitourinary syndrome of menopause	
		Mild	Severe
Epithelial thickness (μm)^+^	503 [467; 550]	261 [244; 289]*^#^	158 [143; 191]*
Boundary of the epithelium and the proper mucous plate	Contrast, wavy	Contrast, smooth	Reduced contrast, smooth
Presence of mucous folds^+^	Yes	No	No
Signal level from the epithelium (RU)^+^	54.1 [51.5; 56.3]	61.6 [55.0; 65.5]	69.7 [67.1; 72.4]*
Signal level from the connective tissue (RU)^+^	70.7 [65.9; 73.7]	79.5 [77.2; 79.9]*^#^	90.32 [80.90; 101.60]*
Stratification index	–0.1284 [–0.1682; –0.1243]	–0.1255 [–0.1644; –0.1088]	–0.1283 [–0.1702; –0.0897]

* Statistically significant difference with the norm; ^#^ statistically significant difference between the mild atrophy group and the severe atrophy group; ^+^ diagnostically significant criteria.

**Figure 6.**
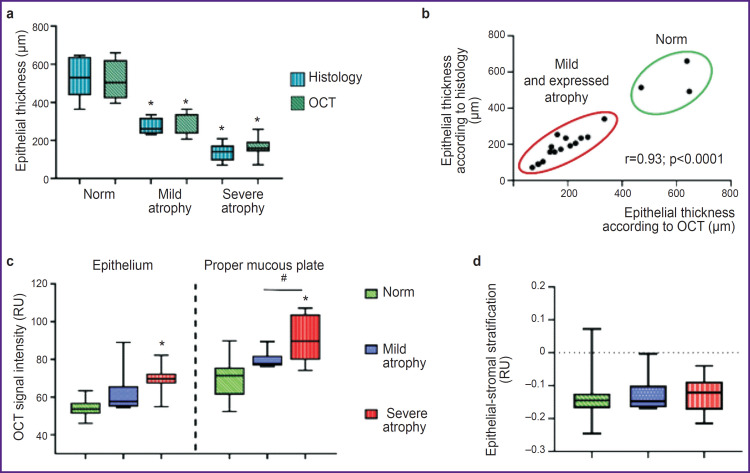
Quantitative assessment of the epithelial thickness, OCT signal intensity in the epithelium and the proper mucous plate, epithelial-stromal stratification The findings are represented in the form of box plots (a)–(c), where the central line is represented by a median, the upper and lower boundaries of the plot body — Q3 and Q1, respectively, “whiskers” — the values within the range of 1.5-fold interquartile range Q1–Q3. A scatter graph (b) reflects high correlation between the measurements made by histological and OCT images; Spearman rank correlation coefficient (r) was used. Statistical analysis was carried out using a nonparametric Mann–Whitley criterion (for paired comparisons) and Kruskal–Wallis test (for multiple comparisons) with Bonferroni adjustment; * groups statistically significantly differed from the norm (p<0.05); ^#^ statistically significant difference of mild and severe atrophy (p<0.05)

The processed data showed significant difference in the epithelial thickness, OCT signal intensity from the epithelium and the proper mucous plate in the women with no vaginal pathology-free and the patients with mild and severe atrophy (Figure 6). Between the groups with mild and severe atrophy there was observed significant difference for the following values: the epithelial thickness and OCT signal intensity from the proper plate. The stratification index made it possible to quantitatively assess the boundary contrast between the epithelium and the proper plate (stroma) of the vaginal wall. Considering the visual contrast decrease in the boundary contrast in atrophy, we supposed the index in atrophy patients to change. However, the calculation of epithelial-stromal stratification (see Figure 6) demonstrated no significant differences between the groups. It was related to the fact that the signal intensity from both: the epithelium and the proper plate (stroma) in vaginal atrophy grows synchronically that would not results in the shift of values to any side, therefore, the stratification does not change. However, here it should be taken into account the small sampling that restricts the statistical estimability.

By now, in literature there have been published the reports on studying the vaginal mucosa by OCT, however, they were focused on describing the technical equipment able to obtain OCT data bulk throughout the vagina [23], or the devices integrated with fractional СО_2_-laser [24]. The pilot study [19] described the vaginal epithelial thickness change in GUSM patients before and after fractional СО_2_-laser therapy. The disadvantage of the mentioned studies is the absence of parallel histological examination and the comparative interpretation of OCT data. It severely restricts medical significance of these works confining them to the masked assessment of the epithelial thickness.

The present study was devoted to the OCT structure assessment of the vaginal mucosa in norm and in GUSM based on the parallel histological examination that enabled to reveal four parameters, which can be used to assess atrophy. According to OCT, insignificant deviations of the epithelial thickness values from those estimated by histological preparations (r=0.93) were found. For the first time there were determined the signal levels from the epithelium and stroma in GUSM and in norm.

Currently, in clinical practice, the vaginal atrophy in GUSM is diagnosed by the past history, a visual estimation, VHI, an epithelial maturation index, colpo- and vaginoscopy; however, modern medicine requires the more objective assessment of the mucosa condition, especially, when studying the feasibility of using new treatment modalities, e.g., non-ablative and fractional lasers [24], as well as for therapy personalization. Noninvasive, rapid and sequential evaluation of the structure serves as an important OCT advantage to assess and compare the efficiency of different therapeutic techniques.

Estrogen level reduction after menopause results in the changes of the connective tissue of the proper plate, the muscular layer, blood vessels, as well as the epithelium [25].

The present study was focused on describing the epithelial changes as the most dynamically and available for studying. For the first time there was described the relationship of the signal level in the epithelium from glycogen accumulated in keratinocytes. Glycogen plays a key role in supporting vaginal microflora and an optimal pH level.

We succeeded in the qualitative and quantitative description of the changes in the connective tissue and the vascular bed. So, the disappearance of the proper plate folds was found in GUSM patients in both: histological study and OCT, and serves as one of the criteria, which occurs even in mild atrophy. The connective tissue change manifested by the increased density and thickness of collagen bundles in the vaginal mucosa and clitoris was described before [25, 26]. However, our study for the first time demonstrated the signal intensity change in structural OCT images related to the connective tissue changes in GUSM.

Marked vascular congestion in a histological examination and the lymphatic bed activation in OCT observed in mild atrophy can be explained by venous stasis. However, despite a marked vasoactive effect of estrogen, which stimulates peripheral circulation by vascular tone reduction [27], the mechanism of venous stasis development in estrogen deficiency requires further explanation.

OCT method is a unique tool able to assess the vaginal epithelial thickness and the epithelial condition. The competitive imaging technique — high-frequency ultrasound (50 MHz) — has insufficient resolution to assess the abovementioned parameters [28], and another a high-resolution method — confocal microscopy — cannot be used intravaginally due to the absence of vaginal probes of an optimal size.

The disadvantage of the present study is a small sampling of women without vaginal pathology (n=3), the limitation is associated with the inability to take biopsy in healthy women.

## Conclusion

Using OCT method there were obtained structural images (B-scans) of the vaginal mucosa, and characterized the optical properties of the tissue both in norm, as well as in mild and severe atrophic mucous changes in terms of genitourinary syndrome of menopause. The study determined 4 criteria, according to which genitourinary syndrome of menopause and its stages can be differentiated: the epithelial thickness, the signal level from the epithelium and the connective tissue, the presence of epithelial folds. In norm, the epithelial tissue was found to be 503 [467; 550] μm, there was revealed the mucous folding; the signal level from the epithelial was 54.1 [51.5; 56.3] RU, while that from the connective tissue — 70.7 [65.9; 73.7] RU. In mild atrophy the study revealed the epithelial thinning up to 261 [244; 289] RU, and no mucous folds, and the signal levels from the epithelium and the connective tissue were 61.6 [55.0; 65.5] and 79.5 [77.2; 79.9] RU, respectively. In severe atrophy the patients were found to have sharp thinning of the mucosa — 158 [143; 191] RU and no folds either; and the signal levels from the epithelium and the connective tissue were 69.7 [67.1; 72.4] and 90.32 [80.90; 101.60] RU, respectively. Atrophy is known to be characterized by glycogen loss and keratohyalin accumulation causing the increasing signal level from the epithelium. Moreover, the signal from the connective tissue increases due to more dense and disordered arrangement of collagen fibers.

Owing to the estimability of the intravital and completely non-invasive assessment of the epithelium and the connective tissue condition, as well as the high rate (nearly in a real-time mode) of OCT scanning, there is the possibility to control over the dynamic changes of the tissue during therapy, and the individual adjustment of a treatment option.
